# A novel mutation of the *CLCN1* gene in a cat with myotonia congenita: Diagnosis and treatment

**DOI:** 10.1111/jvim.16471

**Published:** 2022-07-11

**Authors:** Christian Woelfel, Kathryn Meurs, Steven Friedenberg, Nicole DeBruyne, Natasha J. Olby

**Affiliations:** ^1^ College of Veterinary Medicine North Carolina State University Raleigh North Carolina USA; ^2^ Veterinary Medical Center University of Minnesota Saint Paul Minnesota USA

**Keywords:** chloride channel, electromyography, nondystrophic myotonia, phenytoin

## Abstract

**Case Description:**

A 10‐month‐old castrated male domestic longhair cat was evaluated for increasing frequency of episodic limb rigidity.

**Clinical Findings:**

The cat presented for falling over and lying recumbent with its limbs in extension for several seconds when startled or excited. Upon examination, the cat had hypertrophied musculature, episodes of facial spasm, and a short‐strided, stiff gait.

**Diagnostics:**

Electromyography (EMG) identified spontaneous discharges that waxed and waned in amplitude and frequency, consistent with myotonic discharges. A high impact 8‐base pair (bp) deletion across the end of exon 3 and intron 3 of the chloride voltage‐gated channel 1 (*CLCN1*) gene was identified using whole genome sequencing.

**Treatment and Outcome:**

Phenytoin treatment was initiated at 3 mg/kg po q24 h and resulted in long‐term improvement.

**Clinical Relevance:**

This novel mutation within the *CLCN1* gene is a cause of myotonia congenita in cats and we report for the first time its successful treatment.

**Abbreviations**
b: base pair
CLCN1: chloride voltage‐gated channel 1
DNA: deoxyribonucleic acid
EMG: electromyography
HSF: Human Splicing Finder
Hz: hertz
PCR: polymerase chain reaction
WGS: whole genome sequencing

## 1 INTRODUCTION

Myotonia is a failure of muscle relaxation after voluntary contraction and typically is associated with muscle hypertrophy and stiffness.^1^, ^2^ Disorders causing myotonia are divided into dystrophic and nondystrophic categories.^2^, ^3^, ^4^, ^5^ The nondystrophic myotonias include chloride and sodium channelopathies, and chloride channelopathies, referred to as myotonia congenita, have been reported in many species including mice,^6^ dogs,^1^ cats,^7^, ^8^, ^9^ pigs,^10^ cattle,^11^ horses,^12^, ^13^ goats,^14^, ^15^ and humans.^3^ The first discovery of a mutation within *CLCN1*, a gene that encodes a voltage‐gated chloride channel (ClC‐1), occurred in the goat.^15^ Myotonia congenita was first reported in cats in the late 1990s^7^, ^8^ and, more recently, a mutation in the feline *CLCN1* gene has been reported.^9^ The clinical signs exhibited by cats with myotonia congenita have been described, and treatment has not been reported. Class 1b anti‐arrhythmic drugs that block the sodium channel are the drugs of choice, but cats are very sensitive to this type of treatment. Our purpose was to describe a novel mutation causing myotonia congenita in a cat and to describe both the clinical findings and response to treatment.

## 2 CASE DESCRIPTION

### 2.1 Clinical presentation and diagnostic findings

A 10‐month‐old castrated male domestic longhair cat was presented to North Carolina State Veterinary Hospital for progressively stiff and stilted gait. The cat was adopted by a veterinarian after being surrendered to a local rescue organization at 8 weeks of age because of concerns about its abnormal gait. Initially, the cat had episodes of extending its limbs stiffly for several seconds when first initiating walking, before then returning to a normal gait. These events gradually increased in duration and frequency over the ensuing months and progressed to the cat falling over with all limbs in rigid extension for several seconds during times of activity or surprise (Videos S1 and S2, Supporting Information). Occasionally, the cat would open‐mouth breath during these episodes. The cat had no prior relevant medical history, was on no medications, and was kept indoors.

On physical examination, tachypnea (40 breaths per minute) and dyspnea were present with periods of open‐mouth breathing. The rectal temperature was 103.0°F, and heart rate was within normal limits. Cardiac and pulmonary auscultation were unremarkable. Intermittent blepharospasm in alternating eyes coinciding with lowering of the ipsilateral ear was noted during examination. Epaxial muscle hypertrophy along the cervical and lumbar vertebral column and appendicular muscle hypertrophy of all 4 limbs were appreciated. The cat was ambulatory, with short‐strided and choppy limb movement because of decreased joint flexion. The pelvic limbs were slightly plantigrade and had exaggerated circumduction (decreased adduction). No orthopedic abnormalities were identified, and the remainder of the physical and neurological examinations was unremarkable.

Serum biochemistry disclosed hyperphosphatemia (7.3 mg/dL; reference interval [RI], 2.9‐6.3 mg/dL) and increased alkaline phosphatase activity (81 IU/L; RI, 11‐51 IU/L), consistent with a young growing animal. Creatine kinase activity was within normal limits, as were the results of the CBC. Thoracic radiographs identified a moderately and generally enlarged cardiac silhouette with an absence of generalized overcirculation. The caudal margin of the hepatic silhouette was normal in size but rounded. Cardiac troponin I concentration was within normal limits (<0.2 ng/mL; RI, 0‐0.2 ng/mL). To further investigate the episodic muscle spasms and stiffness, electromyography (EMG) was performed under sedation and identified spontaneous discharges that waxed and waned in amplitude and frequency, consistent with myotonic discharges (Video S3). The frequency of discharges ranged from 40 to 325 Hz, with the majority of discharges ranging between 150 and 250 Hz.

### 2.2 Whole genome sequencing and mutation identification

Approximately 2 mL of EDTA‐anticoagulated blood was collected and DNA was extracted using the standard protocol of the QIAamp DNA Blood Midi Kit from Qiagen (Germantown, Maryland). Approximately 2 μg of DNA from the proband was submitted for Illumina TruSeq polymerase chain reaction (PCR)‐free library preparation and whole genome sequencing (WGS) at Genewiz, LLC (South Plainfield, New Jersey). Detailed methods for the sequencing and variant calling protocols are provided as Data S1. Variants present in the proband were filtered against a database of whole genome sequences derived from 70 cats that have been collected as part of ongoing research in the Veterinary Genetics Laboratory at North Carolina State University. None of the animals in this database were known to have any myopathy, myotonia, or any other similar disorder.

Whole genome sequencing of the proband as described above identified 23 206 variants (biallelic and multiallelic) that were predicted by Variant Effect Predictor (VEP) to have high or moderate impact in at least 1 gene transcript, 280 of which were unique to the proband. High impact variants include splice donor, splice acceptor, frameshift, stop gained, stop lost, and start lost mutations. Moderate impact variants include missense mutations and in‐frame insertions and deletions. Of those unique to the proband, 29 variants were high impact and 251 were moderate impact. Details of these variants and their transcripts are provided in Data S1.

**FIGURE 1:**
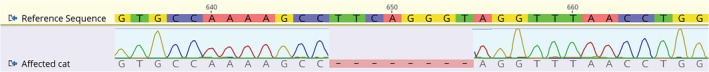
Alignment of the nucleotide sequence from the proband (affected cat) against the reference sequence (UCSC felCat9, top) verifies the presence of an 8 bp deletion at the location chrA2:15897085‐15897092 in chloride voltage‐gated channel 1 (*CLNC1*)

A high impact 8‐base pair (bp) deletion across the end of exon 3 and the beginning of intron 3 of the gene CLCN1 was identified (chrA2:15897085‐15 897 092) in the proband. Sanger sequencing was performed to verify the existence of this 8‐bp deletion (detailed methods are provided in Data S2) and confirmed that the proband was homozygous for the deletion (Figure 1). All phenotypically normal cats in our database of whole genome sequences lacked this deletion. All 96 additional control cats tested by Sanger sequencing were also found to be negative for the deletion.

**FIGURE 2:**
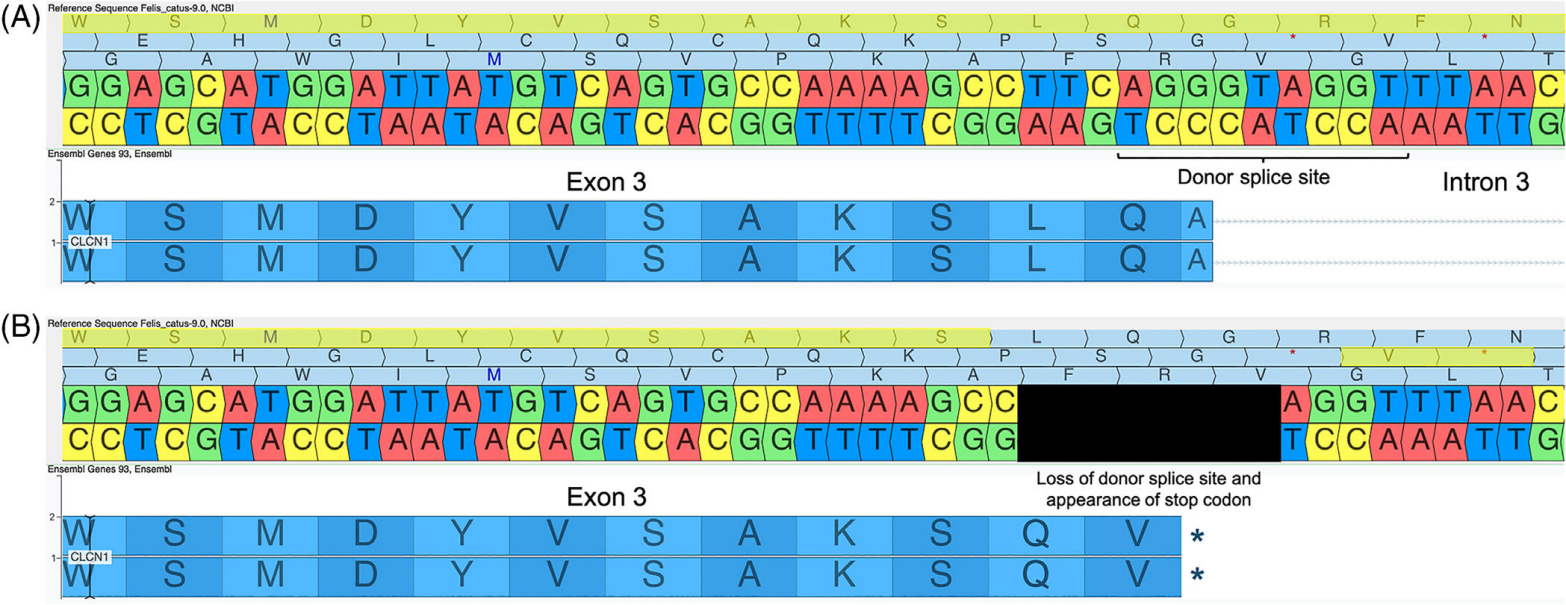
(A) The wild‐type nucleotide and codon sequence at the end of exon 3 and the beginning of intron 3 of feline chloride voltage‐gated channel 1 (*CLNC1*) is shown, with the corresponding reading frame highlighted in yellow. (B) The mutated nucleotide sequence is shown with the deleted nucleotides blacked out, and the adjusted reading frame is highlighted in yellow. The new codon sequence showing the creation of a stop codon is presented below

**FIGURE 3:**
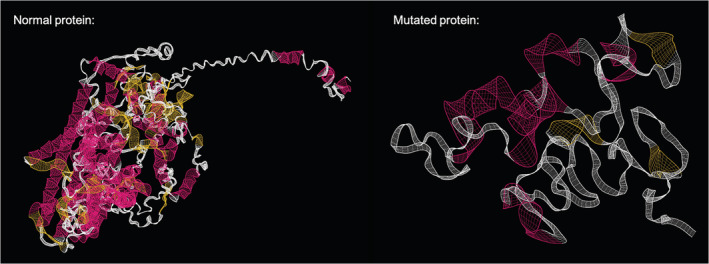
The predicted wild‐type and mutant protein structures of chloride voltage‐gated channel 1 (CLNC1), as modeled by I‐Tasser and visualized in Geneious Prime. The pink color represents alpha helices and yellow represents beta sheets

The effect of the 8‐bp deletion in CLCN1 on alternative splicing was predicted using the Human Splicing Finder (HSF) and on the resulting protein structure was predicted using the I‐Tasser server operated by the Zhang laboratory in the Department of Computational Medicine and Bioinformatics at the University of Michigan and then visualized using Geneious Prime (San Diego, California). In silico analysis of the splice donor site at the junction between exon 3 and intron 3 of CLCN1 using HSF predicted that the donor site is removed in the presence of the 8 bp deletion in question. A decrease in the HSF signal by 12.18% and a lack of MaxEnt Signal at this location in the mutated sequence indicate that this site likely does not serve as a splice donor in the mutated CLCN1 gene. Because of removal of this donor site and the altered reading frame, translation continues beyond the end of exon 3 until it reaches a stop codon normally positioned within the intron (Figure 2). This results in early translation termination and a substantially shorter protein. The typically 23‐exon protein is decreased to only the first 3 exons. The changes in protein structure can be seen in Figure 3.

### 2.3 Treatment and short‐term evaluation

The cat was started on phenytoin at a dosage of 3 mg/kg q 24 h. As reported by the owner, the frequency of myotonic episodes decreased with this medication and the cat was able to run (albeit with a bunny hopping gait) for the first time. However, after 3 months the cat deteriorated with a return to the baseline frequency and severity of episodes and difficulty grooming. On reevaluation at North Carolina State Veterinary Hospital 6 months later, persistent tachypnea was observed, but no episodes of open‐mouth breathing were seen. Blepharospasm and facial spasms were no longer noted. Although the cat remained short‐strided with choppy limb movement and decreased joint flexion in all 4 legs, the pelvic limbs were less plantigrade, less circumduction of these limbs was noted, and the cat was considered to be improved clinically (Video S4). Muscle hypertrophy subjectively was unchanged. The remainder of the neurologic and physical examinations was unremarkable.

Serum biochemistry disclosed persistently increased, but improved, alkaline phosphatase activity (55 IU/L; RI, 11‐51 IU/L) and increased serum globulin concentration (4.7 g/dL; RI, 2.6‐4.5 g/dL). On CBC, a mild nonregenerative anemia (Hct, 30.4%; RI, 33.0‐51.0%) was observed. Plasma protein concentration was increased at 8.4 g/dL (RI, 6.8‐8.3 g/dL). Because of the enlarged cardiac silhouette on previous radiographs, an echocardiogram was performed and showed normal cardiac structure and function. We were unable to find a laboratory to measure serum phenytoin concentration, which therefore could not be monitored. Because of concern about potential toxicity, no changes to medications were made. At follow‐up 1 year later, the owner reported the cat was clinically improved on the same dose of phenytoin.

## 3 DISCUSSION

We identified a novel deletion at the junction of exon 3 and intron 3 of *CLCN1* in a cat with myotonia congenita. This mutation resulted in severe truncation of the CLCN1 chloride channel and resulted in severe clinical signs. Cautious use of phenytoin alleviated clinical signs without adverse effects and the cat was doing well > 1 year after initiation of treatment.

Clinically, myotonic cats can have a stiff and choppy gait, muscle hypertrophy with dimpling after percussion, blepharospasm, facial spasms, dysphonia, dysphagia, and overall may appear unthrifty.^7^, ^8^, ^9^ Our cat shared several of these clinical characteristics, but lacked dimpling with percussion of limb muscles. The tachypnea that was noted on examination was thought to be a response to stress, although failure of relaxation of muscles associated with respiration could have played a role. Interestingly, our cat also had potential cardiac involvement based on the presence of an enlarged heart on radiographs, although echocardiographic evaluation was normal. In a previous report, another myotonic cat had mild enlargement of the left atrium, rounding of the cardiac apex, and pinpoint hyperechoic foci in the left ventricular endocardium on echocardiogram.^7^ Manifestations of nondystrophic myotonia outside of skeletal muscle are rare in humans, because of limited CLCN1 expression in smooth and cardiac muscle.^16^ Furthermore, humans with nondystrophic myotonia and cardiac disease have arrhythmias but not structural heart changes.^17^, ^18^

Diagnosis of myotonia is confirmed using EMG. Needle insertion for EMG causes spontaneous high frequency discharges which have a waxing and waning frequency and amplitude, leading to a characteristic “dive bomber” sound.^19^ The frequency of myotonic discharge in our cat ranged from 40 to 325 Hz, with the majority of discharges being approximately 200 Hz. This frequency is slightly lower than reported previously in cats (mean frequency, 300 Hz),^9^ but higher than that reported for many human patients^20^, ^21^ and, as reported previously in cats, the discharges sounded like a swarm of bees.^9^

Our cat had a high impact 8‐bp deletion across the end of exon 3 and intron 3 of the gene *CLCN1*. Although a mutation within this gene has been documented previously in cats, it was a different mutation, involving exon 16.^9^ Voltage‐gated chloride channels have an important role in maintaining the membrane potential of muscle. These channels facilitate depolarization of an excitable skeletal muscle fiber by allowing an influx of chloride ions.^22^ Therefore, chloride channelopathies decrease chloride ion conductance, and if net chloride conductance is <50% of normal, clinical signs occur.^23^, ^24^ Myotonia congenita can be inherited as an autosomal dominant or recessive trait, with dominant forms causing more severe signs in people, but the genotype‐phenotype correlation is complicated and somewhat difficult to predict.^25^ The ClC‐1 protein is a homodimer formed from both alleles, and in dominant forms of the disease the variant allele can produce a mutant subunit that exhibits a dominant‐negative effect on the wild‐type subunit of the channel, usually by alteration of the voltage dependence of the channel. Recessive forms tend to be protein truncations as found in our cat and in the previously reported cases in cats.^25^ In our case, only the first 3 exons of the 23‐exon gene could be translated, causing a markedly altered protein structure which in turn resulted in prominent clinical signs of myotonia. However, the clinical signs in our cat were consistent with those previously reported in cats with a variant in exon 16.^7^, ^8^, ^9^ In these previously reported cats, although the ClC‐1 protein was less severely truncated, the cystathione β‐synthase cytosolic domain that is critical to ion transport was impacted, as well as the dimerization domain,^9^ both of which also would be impacted in our cat.

Treatment for myotonia in cats has not been previously described. In humans, the preferred treatment for myotonia is mexiletine, which is a class 1B antiarrhythmic drug.^26^ However, this medication can be ineffective or cause clinically relevant adverse effects in patients.^27^ Recently, efforts have been made to address myotonic stiffness by selective blockade of slow sodium channels and increasing potassium ion currents.^27^, ^28^, ^29^, ^30^, ^31^ Treatment was attempted in our cat because of the owner's concern that the myotonic episodes had increased in frequency and seemed distressing to the cat. Phenytoin was chosen because of concerns of adverse effects of class 1A or 1B antiarrhythmic drugs in cats, and given the reported successful treatment of a previous cat with myokymia and neuromyotonia using phenytoin.^32^ Phenytoin is an anticonvulsant medication that stabilizes neuronal membranes by blocking sodium influx and promoting sodium efflux, while also decreasing the sensitivity of muscle spindles to stretch, causing muscle relaxation.^33^, ^34^ With our cat, initial improvement occurred on phenytoin, followed by regression of clinical signs reported by the owner. Yet overall improvement was observed on repeat examination, and on further discussion with the owner, it appeared that signs fluctuated somewhat and that, although the initial marked improvement was not maintained, overall the cat improved with treatment and the owner was satisfied with how the cat was doing 1 year after starting treatment.

Ours is the first report of a *CLCN1* mutation causing a deletion of exon 3 and intron 3 in a young cat with myotonia congenita. Knowledge of different *CLCN1* mutation genotypes is important to determine if correspondingly different phenotypes occur, and this information can allow classification according to respective functional defects, which may help guide treatment. Additionally, ours is the first report of attempted treatment for a cat with myotonia congenita. Phenytoin can be used safely to decrease stiffness and the frequency of myotonic episodes at a dosage of 3 mg/kg PO q24h but long‐term clinical efficacy is uncertain.

## CONFLICT OF INTEREST DECLARATION

Authors declare no conflict of interest.

## OFF‐LABEL ANTIMICROBIAL DECLARATION

Authors declare no off‐label use of antimicrobials.

## INSTITUTIONAL ANIMAL CARE AND USE COMMITTEE (IACUC) OR OTHER APPROVAL DECLARATION

Authors declare no IACUC or other approval was needed.

## HUMAN ETHICS APPROVAL DECLARATION

Authors declare human ethics approval was not needed for this study.

## Supporting information


**Data S1** Detailed methods for whole genome sequencing, sequence alignment, variant picking, Sanger sequencing, and protein prediction.

**Data S2** Details of high impact variants unique to the proband including all transcripts and their impact.

**Video S1** Typical gait of the cat when walking calmly at home. The delayed relaxation of muscle makes the cats gait stiff and mechanical.

**Video S2** When startled by a paper bag the cat becomes recumbent with all limbs extended. Recovery is rapid.

**Video S3** Electromyogram of the cat while under sedation. The classic waxing and waning myotonic discharges can be seen and heard.

**Video S4** Gait of the cat 6 months after initiation of treatment with phenytoin. The gait is still abnormal but the cat is able to move more smoothly than prior to treatment.
