# Supplementary material for: A novel mutation of the CLCN1 gene in a cat with myotonia congenita: Diagnosis and treatment
Source: J Vet Intern Med. 2022 Jul 11;36(4):1454–9. doi: 10.1111/jvim.16471 (PMC9308434; doi:10.1111/jvim.16471)
Supplement: Supplementary file 1 — Data S1 Detailed methods for whole genome sequencing, sequence alignment, variant picking, Sanger sequencing, and protein prediction. [file JVIM-36-1454-s003.pdf]

METHODS

Whole Genome Sequencing: Approximately 2 μg of DNA from the proband was submitted for Illumina TruSeq PCR-free library preparation and whole genome sequencing (WGS) at Genewiz LLC (South Plainfield, NJ). Sequencing experiments were designed as 150 bp paired-end reads and the library was run in one lane of an Illumina HiSeq 4000 high-throughput sequencing system. Variant calling from WGS data was performed using a standardized bioinformatics pipeline essentially as previously described for canines (1). [Briefly, sequence reads were trimmed using Trimmomatic 0.32 (2) to a minimum phred-scaled base quality score of 30 at the start and end of each read with a minimum read length of 70 bp, and aligned to the University of California at Santa Cruz felCat9 reference sequence using BWA 0.7.13 (3). Aligned reads were prepared for analysis using Picard Tools 2.8 (http://broadinstitute.github.io/picard) and GATK 3.7. (4) following best practices for base quality score recalibration and indel realignment specified by the Broad Institute, Cambridge, MA (5,6). Variant calls were made using GATK's HaplotyeCaller walker, and variant quality score recalibration (VQSR) was performed using sites from dbSNP 146 and the Illumina 174K CanineHD BeadChip as training resources. We applied a VQSR tranche sensitivity cutoff of 99.9% to SNPs and 99% to indels for use in downstream analyses; genotype calls with a phred-scaled quality score < 20 were flagged but not removed from the variant callset.]

Variant Filtering and Annotation: Variants (either heterozygous or homozygous) present in the proband were filtered against a database of whole genome sequences derived from 70 cats that have been collected as part of ongoing research in our laboratory. These include 6 Bengals, 5 Domestic Longhair Cats, 37 Domestic Shorthair Cats, 4 Norwegian Forest Cats, 8 Persians, 1 Russian Blue, and 10 Sphynx. Sequence data from all of these cats was processed using the same bioinformatics pipeline described above. None of the animals in this database were known to have any type of myopathy, myotonia, or any other similar disorder. Variants that

passed our filtering step and were unique to the proband were annotated using Variant Effect Predictor 91 and evaluated based on the severity of the predicted effect. Variants considered to be most impactful included frameshifts, in-frame insertions and deletions, premature-start, stop-gained, missense, and splice region changes.

Sanger Sequencing: Based upon the WGS findings, Sanger sequencing was performed to verify the existence of an eight-base pair (bp) deletion across exon 3 and intron 3 of Chloride voltage-gated channel 1 (CLCN1) in the proband. A 235 bp region flanking the deletion of interest was amplified using a polymerase chain reaction (PCR) under standard reaction conditions on a BioRad S1000 thermal cycler using DreamTaq PCR Master Mix (Thermo-Fisher Scientific) with the following primers: F-5'-TGTCTTTCCCTCTTCCCAGA-3' and R-5'-AGCTTATCCCAGGCAGGAAC-3'. The resulting PCR amplicon was sent to Genewiz LLC for Sanger sequencing. This variant was then investigated in an additional 96 control cats of various breeds, composed of 10 Persians, 10 Bengals, 10 Norwegian Forest Cats, 10 Siberian Forest Cats, 10 Maine Coons, 10 Ragdolls, and 26 Domestic Short/Long Haired Cats. All control cats were tested for the CLCN1 using the same protocol described above.

In silico Splice Site Analysis: The effect of the 8bp deletion in CLCN1 on alternative splicing was predicted using Human Splicing Finder (HSF).

Protein Structure Analysis: The effect of the 8bp deletion in CLCN1 on the resulting protein structure was predicted using the I-Tasser server operated by the Zhang lab in the Department of Computational Medicine and Bioinformatics at the University of Michigan and then visualized using Geneious Prime.

1. Friedenberg SG, Meurs KM. Genotype imputation in the domestic dog. *Mamm Genome*. 2016;27:485-94. doi:10.1007/s00335-016-9636-9.
2. Bolger AM, Lohse M, Usadel B. Trimmomatic: a flexible trimmer for Illumina sequence data. *Bioinf*. 2014;30:2114-20. doi:10.1093/bioinformatics/btu170.
3. Li H, Durbin R. Fast and accurate short read alignment with Burrows-Wheeler transform. Bioinformatics. 2009 Jul 2;25(14):1754–60.
4. McKenna A, Hanna M, Banks E, Sivachenko A, Cibulskis K, Kernytsky A, et al. The Genome Analysis Toolkit: A MapReduce framework for analyzing next-generation DNA sequencing data. Genome Res. 2010 Sep 1;20(9):1297–303.
5. DePristo MA, Banks E, Poplin R, Garimella KV, Maguire JR, Hartl C, et al. A framework for variation discovery and genotyping using next-generation DNA sequencing data. Nat Genet. 2011 Apr 10;43(5):491–8.
6. Van der Auwera GA, Carneiro MO, Hartl C, Poplin R, del Angel G, Levy-Moonshine A, et al. From FastQ data to high confidence variant calls: the Genome Analysis Toolkit best practices pipeline. Curr Protoc Bioinformatics. Hoboken, NJ, USA: John Wiley & Sons, Inc; 2013 Oct 15;11(1110):11.10.1–11.10.33.
