# Supplementary material for: A novel mutation of the CLCN1 gene in a cat with myotonia congenita: Diagnosis and treatment
Source: J Vet Intern Med. 2022 Jul 11;36(4):1454–9. doi: 10.1111/jvim.16471 (PMC9308434; doi:10.1111/jvim.16471)
Supplement: Supplementary file 2 — Data S2 Details of high impact variants unique to the proband including all transcripts and their impact. [file JVIM-36-1454-s006.pdf]

Position (felCat9.0)	Ref	Alt	Gene Symbol	ENSEMBL Gene ID	ENSEMBL Transcript ID	Variant Type	Effect
A1:10292960	AC	A	n/a	ENSFCAG00000031325	ENSFCAT00000027051	frameshift variant	HIGH
A1:83744719	C	A	n/a	ENSFCAG00000044316	ENSFCAT00000050411	stop gained	HIGH
A1:86845101	CTTTG	C	n/a	ENSFCAG00000035456	ENSFCAT00000049036	frameshift variant	HIGH
A2:73126183	C	T	n/a	ENSFCAG00000045328	ENSFCAT00000050264	splice acceptor variant & non coding transcript variant	HIGH
A2:158967084	CTTCAGGGT	C	CLCN1	ENSFCAG00000007278	ENSFCAT00000007280	splice donor variant & coding sequence variant	HIGH
					ENSFCAT00000041228	splice donor variant & coding sequence variant	HIGH
A3:24651557	AGC	A	NCOA6	ENSFCAG00000002306	ENSFCAT00000002306	frameshift variant	HIGH
					ENSFCAT00000062029	frameshift variant	HIGH
					ENSFCAT00000065640	frameshift variant	HIGH
A3:92580969	C	T	DUSP11	ENSFCAG00000019224	ENSFCAT00000019207	stop gained	HIGH
			C2orf78	ENSFCAG00000026552	ENSFCAT00000028153	upstream gene variant	MODIFIER
A3:93084265	CTG	C	n/a	ENSFCAG00000008616	ENSFCAT00000008621	splice acceptor variant & intron variant	HIGH
					ENSFCAT00000046912	splice acceptor variant & intron variant	HIGH
					ENSFCAT00000047760	splice acceptor variant & intron variant	HIGH
					ENSFCAT00000052808	splice acceptor variant & intron variant	HIGH
B1:125723765	C	T	STPG2	ENSFCAG00000040357	ENSFCAT00000050738	stop gained	HIGH
B1:187565548	GA	G	ZCCHC4	ENSFCAG00000023396	ENSFCAT00000031715	frameshift variant	HIGH
B4:96602070	T	TG	RAB3IP	ENSFCAG00000015554	ENSFCAT00000015559	intron variant	MODIFIER
			n/a	ENSFCAG00000046521	ENSFCAT00000058555	frameshift variant	HIGH
B4:121638249	AGG	A	RF00563	ENSFCAG00000029370	ENSFCAT00000024452	upstream gene variant	MODIFIER
			SLC25A3	ENSFCAG00000027994	ENSFCAT00000025087	frameshift variant	HIGH
					ENSFCAT00000033040	frameshift variant	HIGH
					ENSFCAT00000062282	frameshift variant	HIGH
B4:121638253	A	ATT	RF00563	ENSFCAG00000029370	ENSFCAT00000024452	upstream gene variant	MODIFIER
			SLC25A3	ENSFCAG00000027994	ENSFCAT00000025087	frameshift variant	HIGH
					ENSFCAT00000033040	frameshift variant	HIGH
					ENSFCAT00000062282	frameshift variant	HIGH
B4:127520915	T	C	n/a	ENSFCAG00000036585	ENSFCAT00000043931	intron variant	MODIFIER
					ENSFCAT00000058001	start lost	HIGH
C1:12054155	C	T	n/a	ENSFCAG00000044602	ENSFCAT00000042892	splice donor variant	HIGH
C1:106226700	GGA	G	SEMA6C	ENSFCAG00000005557	ENSFCAT00000005559	frameshift variant	HIGH
					ENSFCAT00000043019	frameshift variant	HIGH
					ENSFCAT00000053872	3 prime UTR variant	MODIFIER
					ENSFCAT00000060036	downstream gene variant	MODIFIER
					ENSFCAT00000065994	3 prime UTR variant	MODIFIER
C1:107022276	C	T	n/a	ENSFCAG00000041899	ENSFCAT00000057479	start lost	HIGH
C1:194039351	CTT	C	CREB1	ENSFCAG00000008220	ENSFCAT00000008221	frameshift variant	HIGH
					ENSFCAT00000024736	intron variant	MODIFIER
					ENSFCAT00000038569	intron variant	MODIFIER
					ENSFCAT00000061772	frameshift variant	HIGH
C1:195271824	CGCGCGCCG	C	n/a	ENSFCAG00000041864	ENSFCAT00000031236	frameshift variant	HIGH
D1:16618864	C	T	NLRX1	ENSFCAG00000001167	ENSFCAT00000001167	stop gained	HIGH
			PDZD3	ENSFCAG00000001168	ENSFCAT00000001168	upstream gene variant	MODIFIER
					ENSFCAT00000037361	upstream gene variant	MODIFIER
			NLRX1	ENSFCAG00000001167	ENSFCAT00000043500	stop gained	HIGH
D2:33115866	A	AGCTG	TYSND1	ENSFCAG00000044166	ENSFCAT00000060677	downstream gene variant	MODIFIER
					ENSFCAT00000062792	frameshift variant	HIGH
D2:59476548	G	GTGGGTGACAGGAGCTG GGATGGAGGCCTCTGCCA CCAGGGCCCTGTCTCAAT C	PYROXD2	ENSFCAG00000009346	ENSFCAT00000009349	frameshift variant	HIGH
E1:9602491	T	TC	n/a	ENSFCAG00000031027	ENSFCAT00000028891	intron variant	MODIFIER
			PIGL	ENSFCAG00000010824	ENSFCAT00000065686	frameshift variant	HIGH
E1:38746457	CG	C	HOXB3	ENSFCAG00000006408	ENSFCAT00000006410	upstream gene variant	MODIFIER
			MIR10A	ENSFCAG00000016475	ENSFCAT00000016479	downstream gene variant	MODIFIER
			HOXB4	ENSFCAG00000041872	ENSFCAT00000062069	frameshift variant	HIGH
E1:39748436	C	T	GPR179	ENSFCAG00000005812	ENSFCAT00000005815	stop gained	HIGH
E1:51024511	AGGGGCGCCTGGGTGGCGCAGTC GGTTAAGCGTCCGATTTCAGCCAG GTCACGATCTCGCGGTCCGTGAGT TTGAGCCCCGCGTCGGGCTCTGG GCTGATGGCTCAGAGCCTGGAGC CTGTTTCTGATTCTGTGTCTCCCTC TCTCTCTGCCCCTCCCCCGTTCATG CTCTGTCTCTCTCTGTCCCAAAAAT AAATAAATGTTGAAAAAAAAAATT AAAAAAAAAAAAAAAAAAAAGAA ATTCTAAGC	A	n/a	ENSFCAG00000042903	ENSFCAT00000053189	splice donor variant & non coding transcript exon variant & intron variant	HIGH
E2:13446733	T	C	LIPE	ENSFCAG00000022678	ENSFCAT00000026802	stop lost	HIGH
					ENSFCAT00000049512	intron variant	MODIFIER
			n/a	ENSFCAG00000037892	ENSFCAT00000045735	upstream gene variant	MODIFIER
E2:61620435	T	A	n/a	ENSFCAG00000044866	ENSFCAT00000050297	upstream gene variant	MODIFIER

					ENSFCAT00000052013	splice acceptor variant & non coding transcript variant	HIGH
					ENSFCAT00000055872	splice acceptor variant & non coding transcript variant	HIGH
F1:70503299	C	G	DCST1	ENSFCAG00000018847	ENSFCAT00000018172	start lost	HIGH
					ENSFCAT00000058210	start lost	HIGH
			DCST2	ENSFCAG00000040157	ENSFCAT00000053006	intron variant	MODIFIER

Table 1. A list of variants unique to the proband that were called high impact by VEP and the effect in all associated transcripts.

Position (felCat9.0)	Ref	Alt	Gene Symbol	ENSEMBL Gene ID	ENSEMBL Transcript ID	Variant Type	Effect
A1:71234391	A	G	CLYBL	ENSFCAG00000031043	ENSFCAT00000034646	missense variant	MODERATE
					ENSFCAT00000050511	intron variant	MODIFIER
A1:84177713	C	T	n/a	ENSFCAG00000022087	ENSFCAT00000022092	missense variant	MODERATE
A1:91378473	T	A	BTNL9	ENSFCAG00000000991	ENSFCAT00000000991	missense variant	MODERATE
					ENSFCAT00000034165	missense variant	MODERATE
					ENSFCAT00000054663	missense variant	MODERATE
A1:111919100	G	A	SHROOM1	ENSFCAG00000022651	ENSFCAT00000022809	missense variant	MODERATE
					ENSFCAT00000034960	missense variant	MODERATE
A1:113672271	G	T	CAMLG	ENSFCAG00000026813	ENSFCAT00000022858	missense variant	MODERATE
A1:116898026	C	T	CDC25C	ENSFCAG00000031992	ENSFCAT00000028713	missense variant	MODERATE
					ENSFCAT00000055412	missense variant	MODERATE
A1:151761784	G	A	n/a	ENSFCAG00000022528	ENSFCAT00000029163	missense variant	MODERATE
A1:151762007	C	G	n/a	ENSFCAG00000022528	ENSFCAT00000029163	missense variant	MODERATE
A1:159686340	ATCT	A	TTC37	ENSFCAG00000010817	ENSFCAT00000010822	inframe deletion	MODERATE
A1:200642178	T	G	SPINK13	ENSFCAG00000041823	ENSFCAT00000059783	missense variant	MODERATE
A1:208845978	T	C	C7	ENSFCAG00000031466	ENSFCAT00000031719	missense variant	MODERATE
A1:215879699	C	T	ZFR	ENSFCAG00000000354	ENSFCAT00000000354	missense variant	MODERATE
A1:226396297	G	A	CDH18	ENSFCAG00000013641	ENSFCAT00000013645	missense variant	MODERATE
					ENSFCAT00000042027	missense variant	MODERATE
					ENSFCAT00000065136	missense variant	MODERATE
A1:227755754	C	T	n/a	ENSFCAG00000027514	ENSFCAT00000031977	missense variant	MODERATE
A1:228450994	G	A	MYO10	ENSFCAG00000010325	ENSFCAT00000010331	missense variant	MODERATE
					ENSFCAT00000047832	missense variant	MODERATE
A1:234525747	C	T	TAS2R1	ENSFCAG00000012611	ENSFCAT00000012613	missense variant	MODERATE
A2:1898631	G	A	THOP1	ENSFCAG00000015785	ENSFCAT00000015789	missense variant	MODERATE
A2:3447731	T	C	SEMA6B	ENSFCAG00000019404	ENSFCAT00000019041	intron variant	MODIFIER
					ENSFCAT00000063409	missense variant	MODERATE
A2:6705658	G	C	n/a	ENSFCAG00000018988	ENSFCAT00000018708	missense variant & splice region variant	MODERATE
A2:8445900	G	A	LDLR	ENSFCAG00000004895	ENSFCAT00000004896	missense variant	MODERATE
					ENSFCAT00000066000	missense variant	MODERATE
					ENSFCAT00000066710	missense variant	MODERATE
A2:12858503	C	T	CCDC194	ENSFCAG00000046586	ENSFCAT00000056716	missense variant	MODERATE
A2:13640385	C	T	ELL	ENSFCAG00000009739	ENSFCAT00000009741	missense variant	MODERATE
A2:18819664	G	A	UBA7	ENSFCAG00000010680	ENSFCAT00000010683	missense variant	MODERATE
A2:20512376	C	T	n/a	ENSFCAG00000019068	ENSFCAT00000019101	missense variant	MODERATE
A2:20896501	G	C	GLYCTK	ENSFCAG00000015709	ENSFCAT00000015713	missense variant	MODERATE
			MIR135A1	ENSFCAG00000030718	ENSFCAT00000025862	downstream gene variant	MODIFIER
A2:20896867	C	T	GLYCTK	ENSFCAG00000015709	ENSFCAT00000015713	missense variant	MODERATE
			MIR135A1	ENSFCAG00000030718	ENSFCAT00000025862	downstream gene variant	MODIFIER
A2:20947076	G	A	DNAH1	ENSFCAG00000015710	ENSFCAT00000015714	missense variant	MODERATE
A2:20954818	G	A	DNAH1	ENSFCAG00000015710	ENSFCAT00000015714	missense variant	MODERATE
A2:20969580	C	T	DNAH1	ENSFCAG00000015710	ENSFCAT00000015714	missense variant	MODERATE
A2:20975304	C	T	DNAH1	ENSFCAG00000015710	ENSFCAT00000015714	missense variant & splice region variant	MODERATE
A2:20986918	A	G	DNAH1	ENSFCAG00000015710	ENSFCAT00000015714	missense variant	MODERATE
A2:20995868	T	G	DNAH1	ENSFCAG00000015710	ENSFCAT00000015714	missense variant	MODERATE
			BAP1	ENSFCAG00000015711	ENSFCAT00000015715	downstream gene variant	MODIFIER
			n/a	ENSFCAG00000045795	ENSFCAT00000050533	downstream gene variant	MODIFIER
A2:21355829	C	T	ITIH3	ENSFCAG00000015723	ENSFCAT00000015727	missense variant	MODERATE
					ENSFCAT00000051356	downstream gene variant	MODIFIER
					ENSFCAT00000060776	missense variant	MODERATE
			ITIH4	ENSFCAG00000015724	ENSFCAT00000042090	downstream gene variant	MODIFIER
A2:23306612	A	G	CACNA2D3	ENSFCAG00000010121	ENSFCAT00000045267	missense variant	MODERATE
A2:66307090	A	G	SPATA48	ENSFCAG00000038962	ENSFCAT00000043791	missense variant	MODERATE
A2:77886608	T	C	SFRP4	ENSFCAG00000025247	ENSFCAT00000028187	upstream gene variant	MODIFIER
			EPDR1	ENSFCAG00000046351	ENSFCAT00000055170	missense variant	MODERATE
					ENSFCAT00000066100	missense variant	MODERATE
A2:88971202	G	T	PCLO	ENSFCAG00000002901	ENSFCAT00000002902	missense variant	MODERATE
					ENSFCAT00000053548	missense variant	MODERATE
					ENSFCAT00000059654	missense variant	MODERATE
A2:100778052	C	T	DYNC1I1	ENSFCAG00000011808	ENSFCAT00000011811	missense variant	MODERATE
					ENSFCAT00000042395	missense variant	MODERATE
A2:107175563	T	G	VWDE	ENSFCAG00000024923	ENSFCAT00000026113	missense variant	MODERATE
					ENSFCAT00000053776	missense variant	MODERATE
					ENSFCAT00000056388	missense variant	MODERATE
					ENSFCAT00000060638	5 prime UTR variant	MODIFIER
					ENSFCAT00000064584	missense variant	MODERATE
					ENSFCAT00000065663	missense variant	MODERATE
A2:107381020	C	G	SCIN	ENSFCAG00000005088	ENSFCAT00000005091	missense variant	MODERATE
			RF00015	ENSFCAG00000039248	ENSFCAT00000044490	upstream gene variant	MODIFIER
A2:124558814	C	T	ITPRID1	ENSFCAG00000000212	ENSFCAT00000000212	missense variant	MODERATE
					ENSFCAT00000029268	missense variant	MODERATE

A2:124620226	T	A	PPP1R17	ENSFCAG00000033490	ENSFCAT00000047229	missense variant	MODERATE
					ENSFCAT00000052229	missense variant	MODERATE
					ENSFCAT00000054005	missense variant	MODERATE
A2:166065585	C	T	KMT2C	ENSFCAG00000008632	ENSFCAT00000008634	missense variant	MODERATE
A2:166065695	C	T	KMT2C	ENSFCAG00000008632	ENSFCAT00000008634	missense variant	MODERATE
A3:28959300	A	G	n/a	ENSFCAG00000040585	ENSFCAT00000032363	missense variant	MODERATE
					ENSFCAT00000038516	missense variant	MODERATE
A3:56844395	CGCGGTCATG	C	C2orf40	ENSFCAG00000023685	ENSFCAT00000030878	inframe deletion	MODERATE
A3:81535408	C	T	PUS10	ENSFCAG00000008019	ENSFCAT00000008021	missense variant	MODERATE
					ENSFCAT00000052531	missense variant	MODERATE
			n/a	ENSFCAG00000028969	ENSFCAT00000022328	upstream gene variant	MODIFIER
A3:84687830	G	C	AFTPH	ENSFCAG00000028719	ENSFCAT00000023897	missense variant	MODERATE
					ENSFCAT00000048766	missense variant	MODERATE
A3:89709015	G	A	PCYOX1	ENSFCAG00000030129	ENSFCAT00000031948	missense variant	MODERATE
			n/a	ENSFCAG00000045921	ENSFCAT00000058356	downstream gene variant	MODIFIER
					ENSFCAT00000062195	downstream gene variant	MODIFIER
A3:101970247	A	G	DNAH6	ENSFCAG00000000768	ENSFCAT00000000768	missense variant	MODERATE
A3:107592982	C	T	DUSP2	ENSFCAG00000010694	ENSFCAT00000010696	missense variant	MODERATE
B1:1888286	G	A	MYOM2	ENSFCAG00000004727	ENSFCAT00000004734	missense variant	MODERATE
					ENSFCAT00000061368	missense variant	MODERATE
B1:7091214	C	G	n/a	ENSFCAG00000046076	ENSFCAT00000058055	missense variant	MODERATE
B1:17112211	A	G	CYP4V2	ENSFCAG00000001547	ENSFCAT00000001547	missense variant	MODERATE
B1:21265815	G	A	MTMR7	ENSFCAG00000011961	ENSFCAT00000011964	missense variant	MODERATE
B1:21634070	C	A	MICU3	ENSFCAG00000018279	ENSFCAT00000018303	missense variant	MODERATE
					ENSFCAT00000049756	missense variant	MODERATE
B1:42845162	T	C	AP3M2	ENSFCAG00000031044	ENSFCAT00000024267	missense variant	MODERATE
					ENSFCAT00000051861	missense variant	MODERATE
					ENSFCAT00000065406	missense variant	MODERATE
B1:65297575	A	G	TMEM192	ENSFCAG00000038577	ENSFCAT00000037425	missense variant	MODERATE
B1:67245701	G	T	NAF1	ENSFCAG00000031652	ENSFCAT00000061274	missense variant	MODERATE
B1:115410999	C	T	EGF	ENSFCAG00000000506	ENSFCAT00000000506	missense variant	MODERATE
					ENSFCAT00000024046	missense variant	MODERATE
					ENSFCAT00000034737	missense variant	MODERATE
					ENSFCAT00000063173	missense variant	MODERATE
B1:115593791	A	G	n/a	ENSFCAG00000000105	ENSFCAT00000000105	intron variant	MODIFIER
					ENSFCAT00000033262	intron variant	MODIFIER
				ENSFCAG00000034487	ENSFCAT00000034598	upstream gene variant	MODIFIER
					ENSFCAT00000035284	missense variant	MODERATE
					ENSFCAT00000046626	upstream gene variant	MODIFIER
				ENSFCAG00000000105	ENSFCAT00000057565	intron variant	MODIFIER
B1:126201532	A	G	n/a	ENSFCAG00000035537	ENSFCAT00000041970	missense variant	MODERATE
					ENSFCAT00000049515	missense variant	MODERATE
B1:137463098	C	G	n/a	ENSFCAG00000030152	ENSFCAT00000024312	missense variant	MODERATE
B1:138153680	G	A	WDFY3	ENSFCAG00000002946	ENSFCAT00000002946	missense variant	MODERATE
B1:147580951	C	A	AREG	ENSFCAG00000000370	ENSFCAT00000000370	5 prime UTR variant	MODIFIER
					ENSFCAT00000051865	missense variant	MODERATE
B1:203565473	G	A	n/a	ENSFCAG00000046639	ENSFCAT00000066858	missense variant	MODERATE
B1:203703029	G	T	KIAA0232	ENSFCAG00000010024	ENSFCAT00000010026	missense variant	MODERATE
B2:3482796	G	A	n/a	ENSFCAG00000024356	ENSFCAT00000031800	missense variant	MODERATE
B2:11920840	G	T	NUP153	ENSFCAG00000011583	ENSFCAT00000011588	missense variant	MODERATE
					ENSFCAT00000039376	missense variant	MODERATE
			RF00100	ENSFCAG00000038078	ENSFCAT00000047179	downstream gene variant	MODIFIER
B2:18445365	G	A	n/a	ENSFCAG00000038080	ENSFCAT00000040685	missense variant	MODERATE
					ENSFCAT00000058597	missense variant	MODERATE
B2:103086853	T	C	REV3L	ENSFCAG00000028995	ENSFCAT00000025874	missense variant	MODERATE
					ENSFCAT00000053725	missense variant	MODERATE
B2:110042154	C	T	MCM9	ENSFCAG00000028710	ENSFCAT00000026168	missense variant	MODERATE
					ENSFCAT00000036278	intron variant	MODIFIER
B2:125733137	G	A	MTFR2	ENSFCAG00000030038	ENSFCAT00000031085	missense variant	MODERATE
B2:127552387	C	T	ARFGEF3	ENSFCAG00000013904	ENSFCAT00000013910	missense variant	MODERATE
B2:152910321	C	T	UNC93A	ENSFCAG00000006973	ENSFCAT00000006975	missense variant	MODERATE
					ENSFCAT00000045258	missense variant	MODERATE
B3:1157394	C	T	MINAR1	ENSFCAG00000030462	ENSFCAT00000022980	missense variant	MODERATE
B3:2077050	T	C	CEMIP	ENSFCAG00000012937	ENSFCAT00000012941	missense variant	MODERATE
B3:2113570	G	A	CEMIP	ENSFCAG00000012937	ENSFCAT00000012941	missense variant	MODERATE
B3:5551356	C	T	UNC45A	ENSFCAG00000006097	ENSFCAT00000006099	downstream gene variant	MODIFIER
					ENSFCAT00000061787	downstream gene variant	MODIFIER
			RCCD1	ENSFCAG00000006098	ENSFCAT00000006100	missense variant	MODERATE
B3:48283707	C	T	LIPC	ENSFCAG00000008009	ENSFCAT00000008012	missense variant	MODERATE
B3:60524114	T	G	SPATA5L1	ENSFCAG00000019110	ENSFCAT00000019156	missense variant	MODERATE
B3:71343323	G	A	RYR3	ENSFCAG00000000742	ENSFCAT00000000742	missense variant	MODERATE
					ENSFCAT00000027046	missense variant	MODERATE
B3:76288090	C	T	THTPA	ENSFCAG00000012059	ENSFCAT00000012062	downstream gene variant	MODIFIER
			JPH4	ENSFCAG00000018964	ENSFCAT00000018381	downstream gene variant	MODIFIER
			AP1G2	ENSFCAG00000040037	ENSFCAT00000042508	missense variant	MODERATE

B3:99771839	A	G	KLHDC1	ENSFCAG00000036283	ENSFCAT00000038314	missense variant	MODERATE
B3:99820836	C	G	NEMF	ENSFCAG00000018946	ENSFCAT00000019170	missense variant	MODERATE
			KLHDC2	ENSFCAG00000031902	ENSFCAT00000028217	downstream gene variant	MODIFIER
					ENSFCAT00000037457	downstream gene variant	MODIFIER
B3:100769651	G	T	ABHD12B	ENSFCAG00000002777	ENSFCAT00000002777	downstream gene variant	MODIFIER
			PYGL	ENSFCAG00000002778	ENSFCAT00000002778	missense variant	MODERATE
					ENSFCAT00000040133	missense variant	MODERATE
					ENSFCAT00000048003	missense variant	MODERATE
B3:124433403	C	T	TMED8	ENSFCAG00000011732	ENSFCAT00000011734	missense variant	MODERATE
B3:124439476	G	T	TMED8	ENSFCAG00000011732	ENSFCAT00000011734	missense variant	MODERATE
B3:124468274	T	C	NOXRED1	ENSFCAG00000039154	ENSFCAT00000046884	downstream gene variant	MODIFIER
			n/a	ENSFCAG00000042664	ENSFCAT00000060305	missense variant	MODERATE
B3:124719696	A	T	ALKBH1	ENSFCAG00000037897	ENSFCAT00000037675	missense variant	MODERATE
			SLIRP	ENSFCAG00000036326	ENSFCAT00000039204	upstream gene variant	MODIFIER
B3:124734389	C	T	SNW1	ENSFCAG00000001637	ENSFCAT00000001637	intron variant	MODIFIER
			SLIRP	ENSFCAG00000036326	ENSFCAT00000039204	missense variant & splice region variant	MODERATE
B3:136530564	A	G	TTC7B	ENSFCAG00000001583	ENSFCAT00000001583	missense variant	MODERATE
					ENSFCAT00000058981	missense variant	MODERATE
					ENSFCAT00000063691	missense variant	MODERATE
					ENSFCAT00000067224	missense variant	MODERATE
B3:139320529	C	T	DDX24	ENSFCAG00000010229	ENSFCAT00000010232	missense variant	MODERATE
					ENSFCAT00000065890	missense variant	MODERATE
B3:139329845	C	T	DDX24	ENSFCAG00000010229	ENSFCAT00000010232	missense variant	MODERATE
					ENSFCAT00000065890	missense variant	MODERATE
B3:140225968	C	T	DICER1	ENSFCAG00000003535	ENSFCAT00000003537	missense variant	MODERATE
					ENSFCAT00000037515	missense variant	MODERATE
B4:5397248	G	A	n/a	ENSFCAG00000009007	ENSFCAT00000009009	missense variant	MODERATE
B4:25101091	G	A	MASTL	ENSFCAG00000014240	ENSFCAT00000014244	missense variant	MODERATE
					ENSFCAT00000034455	missense variant	MODERATE
B4:46708262	T	G	KLRK1	ENSFCAG00000040686	ENSFCAT00000053631	missense variant	MODERATE
B4:46708276	T	C	KLRK1	ENSFCAG00000040686	ENSFCAT00000053631	missense variant	MODERATE
B4:46993758	T	C	n/a	ENSFCAG00000025057	ENSFCAT00000022489	missense variant & splice region variant	MODERATE
					ENSFCAT00000050581	missense variant & splice region variant	MODERATE
B4:81064790	A	G	KRT74	ENSFCAG00000000290	ENSFCAT00000000288	missense variant	MODERATE
					ENSFCAT00000050616	missense variant	MODERATE
B4:81072162	A	C	KRT74	ENSFCAG00000000290	ENSFCAT00000000288	missense variant	MODERATE
					ENSFCAT00000050616	missense variant	MODERATE
B4:81164526	A	G	KRT4	ENSFCAG00000000295	ENSFCAT00000000295	intron variant	MODIFIER
			KRT1	ENSFCAG00000042595	ENSFCAT00000055089	missense variant	MODERATE
B4:82671186	C	T	ZNF385A	ENSFCAG00000010154	ENSFCAT00000010157	missense variant	MODERATE
					ENSFCAT00000053337	intron variant	MODIFIER
B4:115206387	G	A	LUM	ENSFCAG00000003764	ENSFCAT00000003763	missense variant	MODERATE
					ENSFCAT00000033907	missense variant	MODERATE
B4:126019306	G	A	STAB2	ENSFCAG00000007290	ENSFCAT00000007294	missense variant	MODERATE
B4:126124621	G	A	STAB2	ENSFCAG00000007290	ENSFCAT00000007294	missense variant	MODERATE
B4:126578829	G	A	TXNRD1	ENSFCAG00000003645	ENSFCAT00000003645	intron variant	MODIFIER
					ENSFCAT00000044250	missense variant	MODERATE
B4:127520710	T	G	n/a	ENSFCAG00000036585	ENSFCAT00000043931	missense variant	MODERATE
					ENSFCAT00000058001	intron variant	MODIFIER
B4:127520870	T	C	n/a	ENSFCAG00000036585	ENSFCAT00000043931	missense variant & splice region variant	MODERATE
				ENSFCAG00000036585	ENSFCAT00000058001	missense variant & splice region variant	MODERATE
B4:127524132	C	G	n/a	ENSFCAG00000036585	ENSFCAT00000043931	missense variant	MODERATE
					ENSFCAT00000058001	upstream gene variant	MODIFIER
C1:1984637	G	A	MEGF6	ENSFCAG00000031840	ENSFCAT00000032322	missense variant	MODERATE
					ENSFCAT00000048790	missense variant	MODERATE
C1:7687386	C	T	CASZ1	ENSFCAG00000014593	ENSFCAT00000014597	missense variant	MODERATE
					ENSFCAT00000054128	missense variant	MODERATE
					ENSFCAT00000058061	missense variant	MODERATE
C1:18305859	G	A	NIPAL3	ENSFCAG00000002067	ENSFCAT00000002067	downstream gene variant	MODIFIER
					ENSFCAT00000036295	missense variant	MODERATE
C1:24065622	T	C	COL16A1	ENSFCAG00000015669	ENSFCAT00000015679	intron variant	MODIFIER
					ENSFCAT00000044147	missense variant	MODERATE
C1:30550610	C	T	MACF1	ENSFCAG00000045633	ENSFCAT00000044017	missense variant	MODERATE
					ENSFCAT00000055061	missense variant	MODERATE
					ENSFCAT00000055111	missense variant	MODERATE
					ENSFCAT00000064822	missense variant	MODERATE
C1:42573761	G	A	TUT4	ENSFCAG00000003246	ENSFCAT00000003246	missense variant	MODERATE
C1:51611279	T	C	DOCK7	ENSFCAG00000022720	ENSFCAT00000032284	missense variant	MODERATE
					ENSFCAT00000048381	missense variant	MODERATE
C1:73288084	G	A	CLCA2	ENSFCAG00000008210	ENSFCAT00000008212	missense variant	MODERATE

C1:104413373	C	T	PDZK1	ENSFCAG00000013368	ENSFCAT00000013371	missense variant	MODERATE
					ENSFCAT00000045829	missense variant	MODERATE
			n/a	ENSFCAG00000024477	ENSFCAT00000027219	downstream gene variant	MODIFIER
C1:107100857	T	G	TCHHL1	ENSFCAG00000046571	ENSFCAT00000052456	missense variant	MODERATE
C1:113656609	A	G	MYO7B	ENSFCAG00000013176	ENSFCAT00000013180	missense variant	MODERATE
					ENSFCAT00000036804	intron variant	MODIFIER
					ENSFCAT00000038750	intron variant	MODIFIER
C1:113705761	C	A	IWS1	ENSFCAG00000009026	ENSFCAT00000009028	missense variant	MODERATE
					ENSFCAT00000037913	intron variant	MODIFIER
C1:154085770	G	A	IFIH1	ENSFCAG00000023951	ENSFCAT00000007305	missense variant	MODERATE
					ENSFCAT00000026735	missense variant	MODERATE
					ENSFCAT00000061411	missense variant	MODERATE
C1:161995864	GGCCGCCGCC	G	SP5	ENSFCAG00000044347	ENSFCAT00000050366	inframe deletion	MODERATE
C1:164281618	G	A	n/a	ENSFCAG00000008059	ENSFCAT00000054583	missense variant	MODERATE
C1:164350082	T	C	CDCA7	ENSFCAG00000005476	ENSFCAT00000005479	missense variant	MODERATE
					ENSFCAT00000042357	missense variant	MODERATE
					ENSFCAT00000050183	missense variant	MODERATE
C1:165241721	G	GGAA	CIR1	ENSFCAG00000022845	ENSFCAT00000030886	inframe insertion	MODERATE
C1:165593144	A	T	CHRNA1	ENSFCAG00000014103	ENSFCAT00000014107	missense variant	MODERATE
					ENSFCAT00000054627	missense variant	MODERATE
					ENSFCAT00000065105	missense variant	MODERATE
C1:169226811	G	A	n/a	ENSFCAG00000046559	ENSFCAT00000054332	missense variant	MODERATE
					ENSFCAT00000057628	missense variant	MODERATE
C1:195274259	G	A	n/a	ENSFCAG00000041864	ENSFCAT00000031236	missense variant	MODERATE
C1:203392796	T	C	AAMP	ENSFCAG00000002372	ENSFCAT00000002372	upstream gene variant	MODIFIER
					ENSFCAT00000053285	upstream gene variant	MODIFIER
			TMBIM1	ENSFCAG00000002374	ENSFCAT00000002374	downstream gene variant	MODIFIER
			n/a	ENSFCAG00000040250	ENSFCAT00000053237	missense variant	MODERATE
C1:219283421	C	T	COL6A3	ENSFCAG00000012720	ENSFCAT00000012724	missense variant	MODERATE
					ENSFCAT00000051285	missense variant	MODERATE
					ENSFCAT00000052409	missense variant	MODERATE
					ENSFCAT00000056790	missense variant	MODERATE
					ENSFCAT00000063857	missense variant	MODERATE
C1:220011772	A	G	n/a	ENSFCAG00000046551	ENSFCAT00000052753	missense variant	MODERATE
C1:221728541	G	C	ANKMY1	ENSFCAG00000012464	ENSFCAT00000012469	missense variant	MODERATE
					ENSFCAT00000036897	missense variant	MODERATE
					ENSFCAT00000037799	missense variant	MODERATE
					ENSFCAT00000039736	missense variant	MODERATE
					ENSFCAT00000040710	missense variant	MODERATE
C1:221768848	C	T	CAPN10	ENSFCAG00000012470	ENSFCAT00000012473	missense variant	MODERATE
					ENSFCAT00000036494	missense variant	MODERATE
					ENSFCAT00000063353	intron variant	MODIFIER
					ENSFCAT00000063602	intron variant	MODIFIER
C1:221791166	C	T	GPR35	ENSFCAG00000024715	ENSFCAT00000027678	missense variant	MODERATE
C2:650249	G	A	SPATC1L	ENSFCAG00000014816	ENSFCAT00000014820	missense variant	MODERATE
C2:1931874	G	GTGCAGCACACGGGCT	TSPEAR	ENSFCAG00000001772	ENSFCAT00000001772	intron variant	MODIFIER
			n/a	ENSFCAG00000035693	ENSFCAT00000032850	inframe insertion	MODERATE
				ENSFCAG00000033709	ENSFCAT00000034604	downstream gene variant	MODIFIER
C2:2111583	C	T	TRPM2	ENSFCAG00000009173	ENSFCAT00000009175	missense variant	MODERATE
					ENSFCAT00000056225	missense variant	MODERATE
					ENSFCAT00000060156	missense variant	MODERATE
			RF00285	ENSFCAG00000035800	ENSFCAT00000033694	downstream gene variant	MODIFIER
				ENSFCAG00000034468	ENSFCAT00000036764	downstream gene variant	MODIFIER
C2:2117668	GTCC	G	TRPM2	ENSFCAG00000009173	ENSFCAT00000009175	inframe deletion	MODERATE
					ENSFCAT00000056225	inframe deletion	MODERATE
					ENSFCAT00000060156	inframe deletion	MODERATE
			RF00285	ENSFCAG00000035800	ENSFCAT00000033694	upstream gene variant	MODIFIER
				ENSFCAG00000034468	ENSFCAT00000036764	upstream gene variant	MODIFIER
C2:2170115	G	C	CFAP410	ENSFCAG00000040115	ENSFCAT00000040416	missense variant	MODERATE
					ENSFCAT00000059030	missense variant	MODERATE
C2:2359138	C	T	n/a	ENSFCAG00000004487	ENSFCAT00000004491	missense variant	MODERATE
					ENSFCAT00000037759	missense variant	MODERATE
				ENSFCAG00000008440	ENSFCAT00000054406	downstream gene variant	MODIFIER
C2:8372249	C	T	VPS26C	ENSFCAG00000012830	ENSFCAT00000012834	missense variant	MODERATE
					ENSFCAT00000047540	missense variant	MODERATE
					ENSFCAT00000050115	missense variant	MODERATE
					ENSFCAT00000060256	missense variant	MODERATE
C2:48628614	G	A	TFG	ENSFCAG00000025659	ENSFCAT00000027637	missense variant	MODERATE
					ENSFCAT00000050806	missense variant	MODERATE
					ENSFCAT00000059364	missense variant	MODERATE
C2:55837148	G	A	RETNLB	ENSFCAG00000036016	ENSFCAT00000049101	missense variant	MODERATE
C2:84089300	C	A	SENP2	ENSFCAG00000042538	ENSFCAT00000055178	missense variant	MODERATE
C2:95840959	G	A	NCEH1	ENSFCAG00000028457	ENSFCAT00000024541	missense variant	MODERATE
C2:107223987	C	A	IL12A	ENSFCAG00000011130	ENSFCAT00000011133	missense variant	MODERATE
C2:116164833	C	T	TSC22D2	ENSFCAG00000031850	ENSFCAT00000023131	missense variant	MODERATE
					ENSFCAT00000047483	missense variant	MODERATE

C2:156182225	G	A	DCLK3	ENSFCAG00000022392	ENSFCAT00000032249	missense variant	MODERATE
					ENSFCAT00000063849	missense variant	MODERATE
D1:1515464	C	T	MMP13	ENSFCAG00000000118	ENSFCAT00000000118	missense variant	MODERATE
D1:12166851	G	A	NNMT	ENSFCAG00000037839	ENSFCAT00000037849	missense variant	MODERATE
D1:16618344	G	A	NLRX1	ENSFCAG00000001167	ENSFCAT00000001167	missense variant	MODERATE
					ENSFCAT00000043500	missense variant	MODERATE
			PDZD3	ENSFCAG00000001168	ENSFCAT00000001168	upstream gene variant	MODIFIER
					ENSFCAT00000037361	upstream gene variant	MODIFIER
D1:16627280	G	A	PDZD3	ENSFCAG00000001168	ENSFCAT00000001168	downstream gene variant	MODIFIER
					ENSFCAT00000037361	downstream gene variant	MODIFIER
			CCDC153	ENSFCAG00000001169	ENSFCAT00000001169	missense variant	MODERATE
D1:16629401	C	T	PDZD3	ENSFCAG00000001168	ENSFCAT00000001168	downstream gene variant	MODIFIER
					ENSFCAT00000037361	downstream gene variant	MODIFIER
			CCDC153	ENSFCAG00000001169	ENSFCAT00000001169	missense variant	MODERATE
D1:23231738	A	T	RPUSD4	ENSFCAG00000029789	ENSFCAT00000022983	missense variant	MODERATE
D1:40592747	A	G	SESN3	ENSFCAG00000005081	ENSFCAT00000005082	missense variant	MODERATE
					ENSFCAT00000056681	missense variant	MODERATE
					ENSFCAT00000057835	missense variant	MODERATE
D1:61137254	C	CCCCCGG	ARAP1	ENSFCAG00000013192	ENSFCAT00000013196	protein altering variant	MODERATE
					ENSFCAT00000055940	protein altering variant	MODERATE
D1:89038010	A	G	QSER1	ENSFCAG00000015204	ENSFCAT00000015210	missense variant	MODERATE
					ENSFCAT00000040727	missense variant	MODERATE
					ENSFCAT00000062010	missense variant	MODERATE
D1:109146850	A	AGGC	RTN3	ENSFCAG00000023202	ENSFCAT00000024505	downstream gene variant	MODIFIER
					ENSFCAT00000066093	downstream gene variant	MODIFIER
			C11orf95	ENSFCAG00000032639	ENSFCAT00000033573	inframe insertion	MODERATE
					ENSFCAT00000055356	downstream gene variant	MODIFIER
D1:110362164	C	T	CAPN1	ENSFCAG00000001438	ENSFCAT00000001438	missense variant	MODERATE
					ENSFCAT00000058310	missense variant	MODERATE
D1:113819532	G	T	CCND1	ENSFCAG00000008358	ENSFCAT00000008360	missense variant	MODERATE
					ENSFCAT00000058874	downstream gene variant	MODIFIER
D2:6677819	C	A	A1CF	ENSFCAG00000011179	ENSFCAT00000011182	missense variant	MODERATE
					ENSFCAT00000067045	missense variant	MODERATE
D2:8660764	A	C	FAS	ENSFCAG00000033129	ENSFCAT00000036758	missense variant	MODERATE
D2:62911000	C	T	ACTR1A	ENSFCAG00000001248	ENSFCAT00000001248	missense variant	MODERATE
					ENSFCAT00000058888	downstream gene variant	MODIFIER
			MFSD13A	ENSFCAG00000023881	ENSFCAT00000028865	downstream gene variant	MODIFIER
D2:64308953	C	T	COL17A1	ENSFCAG00000004299	ENSFCAT00000004301	missense variant	MODERATE
					ENSFCAT00000046187	missense variant	MODERATE
D3:19138629	C	A	GSTT4	ENSFCAG00000036794	ENSFCAT00000045313	missense variant	MODERATE
D3:19160731	C	A	n/a	ENSFCAG00000022623	ENSFCAT00000022132	downstream gene variant	MODIFIER
				ENSFCAG00000035422	ENSFCAT00000040173	missense variant	MODERATE
D3:55423196	G	C	DSG2	ENSFCAG00000031128	ENSFCAT00000023465	missense variant	MODERATE
D3:55423210	G	A	DSG2	ENSFCAG00000031128	ENSFCAT00000023465	missense variant	MODERATE
D4:11831268	C	T	n/a	ENSFCAG00000008814	ENSFCAT00000008816	missense variant	MODERATE
					ENSFCAT00000058936	missense variant	MODERATE
					ENSFCAT00000060196	missense variant	MODERATE
					ENSFCAT00000061958	missense variant	MODERATE
					ENSFCAT00000064926	missense variant	MODERATE
D4:17623266	G	A	PCSK5	ENSFCAG00000027896	ENSFCAT00000024077	missense variant	MODERATE
					ENSFCAT00000050249	missense variant	MODERATE
					ENSFCAT00000059461	5 prime UTR variant	MODIFIER
D4:18650803	GGCGGCCGCGGCGGTGGGGAGCGTGGGGCGCCTGTCGCAGTTCCCGCCCTACGGGTTGGGCTCCGCCGCCGCGGCCGCCGCGGCC	G	FOXB2	ENSFCAG00000046576	ENSFCAT00000056177	inframe deletion	MODERATE
D4:29961035	T	C	n/a	ENSFCAG00000002729	ENSFCAT00000002729	missense variant	MODERATE
D4:61261685	G	C	FRMPD1	ENSFCAG00000011920	ENSFCAT00000011923	missense variant	MODERATE
			TRMT10B	ENSFCAG00000035239	ENSFCAT00000040640	upstream gene variant	MODIFIER
D4:61380631	A	G	n/a	ENSFCAG00000036372	ENSFCAT00000036949	missense variant	MODERATE
D4:81633846	G	A	CDK5RAP2	ENSFCAG00000008625	ENSFCAT00000008629	missense variant	MODERATE
					ENSFCAT00000054488	missense variant	MODERATE
					ENSFCAT00000057225	missense variant	MODERATE
D4:81969996	G	A	PSMD5	ENSFCAG00000001313	ENSFCAT00000001312	downstream gene variant	MODIFIER
					ENSFCAT00000057489	downstream gene variant	MODIFIER
			B3GNT10	ENSFCAG00000022327	ENSFCAT00000029226	missense variant	MODERATE

D4:91123222	G	C	LAMC3	ENSFCAG00000005118	ENSFCAT00000005119	missense variant	MODERATE
E1:14535925	A	T	MNT	ENSFCAG00000006315	ENSFCAT00000035165	missense variant	MODERATE
					ENSFCAT00000058918	downstream gene variant	MODIFIER
E1:15201590	G	A	SCARF1	ENSFCAG00000044300	ENSFCAT00000062099	missense variant	MODERATE
E1:16753714	C	T	NSRP1	ENSFCAG00000039697	ENSFCAT00000035282	missense variant	MODERATE
E1:16968924	C	T	EFCAB5	ENSFCAG00000004649	ENSFCAT00000004651	missense variant	MODERATE
			n/a	ENSFCAG00000044178	ENSFCAT00000057662	upstream gene variant	MODIFIER
E1:17328108	C	T	ABHD15	ENSFCAG00000002756	ENSFCAT00000002756	upstream gene variant	MODIFIER
			TP53I13	ENSFCAG00000002757	ENSFCAT00000002757	missense variant	MODERATE
			GIT1	ENSFCAG00000002758	ENSFCAT00000042777	downstream gene variant	MODIFIER
					ENSFCAT00000047071	downstream gene variant	MODIFIER
E1:30545053	C	T	TSPOAP1	ENSFCAG00000012264	ENSFCAT00000012268	missense variant	MODERATE
					ENSFCAT00000044954	missense variant	MODERATE
E1:30636189	G	A	MKS1	ENSFCAG00000012261	ENSFCAT00000012264	missense variant	MODERATE
					ENSFCAT00000052159	missense variant	MODERATE
			n/a	ENSFCAG00000034800	ENSFCAT00000033967	downstream gene variant	MODIFIER
E1:30682939	G	A	OR4D1	ENSFCAG00000044467	ENSFCAT00000030441	missense variant	MODERATE
E1:31056420	C	T	CCDC182	ENSFCAG00000001699	ENSFCAT00000001699	missense variant	MODERATE
E1:36539075	C	T	MBTD1	ENSFCAG00000006743	ENSFCAT00000006745	upstream gene variant	MODIFIER
					ENSFCAT00000041488	upstream gene variant	MODIFIER
			UTP18	ENSFCAG00000031396	ENSFCAT00000029778	missense variant	MODERATE
					ENSFCAT00000030760	missense variant	MODERATE
E1:37265200	G	A	EME1	ENSFCAG00000012332	ENSFCAT00000012335	downstream gene variant	MODIFIER
			LRRC59	ENSFCAG00000031770	ENSFCAT00000022309	missense variant	MODERATE
E1:41039872	G	A	MED24	ENSFCAG00000014993	ENSFCAT00000014997	intron variant	MODIFIER
					ENSFCAT00000041870	missense variant	MODERATE
					ENSFCAT00000043432	intron variant	MODIFIER
					ENSFCAT00000046079	intron variant	MODIFIER
					ENSFCAT00000051893	intron variant	MODIFIER
			RF01211	ENSFCAG00000033090	ENSFCAT00000034933	downstream gene variant	MODIFIER
			CSF3	ENSFCAG00000040124	ENSFCAT00000043767	downstream gene variant	MODIFIER
					ENSFCAT00000063974	downstream gene variant	MODIFIER
E1:41147641	A	G	CASC3	ENSFCAG00000014998	ENSFCAT00000015002	missense variant	MODERATE
E1:44190339	G	C	HDAC5	ENSFCAG00000002199	ENSFCAT00000002199	missense variant	MODERATE
					ENSFCAT00000036901	missense variant	MODERATE
E1:44239695	G	C	C17orf53	ENSFCAG00000008021	ENSFCAT00000008023	missense variant & splice region variant	MODERATE
					ENSFCAT00000065449	intron variant	MODIFIER
E1:44726300	G	A	DBF4B	ENSFCAG00000023490	ENSFCAT00000023498	missense variant	MODERATE
					ENSFCAT00000060046	missense variant	MODERATE
E1:45347104	C	A	n/a	ENSFCAG00000046546	ENSFCAT00000053446	missense variant	MODERATE
					ENSFCAT00000061422	intron variant	MODIFIER
E1:45347109	G	A	n/a	ENSFCAG00000046546	ENSFCAT00000053446	missense variant	MODERATE
					ENSFCAT00000061422	intron variant	MODIFIER
E1:45347329	C	T	n/a	ENSFCAG00000046546	ENSFCAT00000053446	missense variant	MODERATE
					ENSFCAT00000061422	intron variant	MODIFIER
E1:61100712	C	T	RNF213	ENSFCAG00000000945	ENSFCAT00000000947	missense variant	MODERATE
					ENSFCAT00000040505	missense variant	MODERATE
E1:61769120	C	T	TEPSIN	ENSFCAG00000007574	ENSFCAT00000007575	missense variant	MODERATE
E2:391338	G	A	ZNF132	ENSFCAG00000034735	ENSFCAT00000037402	missense variant	MODERATE
E2:3164628	C	G	SBK2	ENSFCAG00000018172	ENSFCAT00000019391	missense variant	MODERATE
E2:3275072	C	T	n/a	ENSFCAG00000005639	ENSFCAT00000005641	upstream gene variant	MODIFIER
			TMEM238	ENSFCAG00000042544	ENSFCAT00000051974	missense variant	MODERATE
			TMEM190	ENSFCAG00000044046	ENSFCAT00000058607	downstream gene variant	MODIFIER
E2:3296678	C	G	n/a	ENSFCAG00000005637	ENSFCAT00000027091	missense variant	MODERATE
			COX6B2	ENSFCAG00000045733	ENSFCAT00000054765	upstream gene variant	MODIFIER
					ENSFCAT00000062952	upstream gene variant	MODIFIER
E2:4510569	A	C	n/a	ENSFCAG00000000122	ENSFCAT00000000122	missense variant	MODERATE
E2:7229279	C	T	n/a	ENSFCAG00000002858	ENSFCAT00000000274	missense variant	MODERATE
					ENSFCAT00000002858	missense variant	MODERATE
					ENSFCAT00000013695	missense variant	MODERATE
E2:7279785	T	C	n/a	ENSFCAG00000046174	ENSFCAT00000063304	missense variant	MODERATE
E2:10578436	G	A	PTGIR	ENSFCAG00000027533	ENSFCAT00000031423	missense variant	MODERATE
E2:11383116	A	T	PPP1R13L	ENSFCAG00000033518	ENSFCAT00000033821	missense variant	MODERATE
E2:12882208	G	A	LYPD3	ENSFCAG00000025558	ENSFCAT00000022021	missense variant	MODERATE
E2:12980296	C	G	n/a	ENSFCAG00000042962	ENSFCAT00000050319	missense variant	MODERATE
E2:12980375	C	A	n/a	ENSFCAG00000042962	ENSFCAT00000050319	missense variant	MODERATE
E2:12980725	T	C	n/a	ENSFCAG00000042962	ENSFCAT00000050319	missense variant	MODERATE
E2:13013310	T	C	n/a	ENSFCAG00000044759	ENSFCAT00000053023	missense variant	MODERATE
E2:13163878	T	C	GRIK5	ENSFCAG00000012730	ENSFCAT00000012733	missense variant	MODERATE

					ENSFCAT00000055225	missense variant	MODERATE
					ENSFCAT00000057632	missense variant	MODERATE
					ENSFCAT00000062684	missense variant	MODERATE
					ENSFCAT00000063192	missense variant	MODERATE
					ENSFCAT00000065353	missense variant	MODERATE
E2:13328101	C	T	ERF	ENSFCAG00000030101	ENSFCAT00000029651	missense variant	MODERATE
E2:32289680	C	T	n/a	ENSFCAG00000030405	ENSFCAT00000028957	missense variant	MODERATE
E2:36806817	A	G	KATNB1	ENSFCAG00000002687	ENSFCAT00000002687	missense variant	MODERATE
			KIFC3	ENSFCAG00000002689	ENSFCAT00000035824	downstream gene variant	MODIFIER
					ENSFCAT00000037293	downstream gene variant	MODIFIER
					ENSFCAT00000045877	downstream gene variant	MODIFIER
					ENSFCAT00000051211	downstream gene variant	MODIFIER
E3:774328	T	C	DNAAF5	ENSFCAG00000005248	ENSFCAT00000038033	missense variant	MODERATE
E3:797033	C	T	DNAAF5	ENSFCAG00000005248	ENSFCAT00000038033	missense variant	MODERATE
E3:797036	G	A	DNAAF5	ENSFCAG00000005248	ENSFCAT00000038033	missense variant	MODERATE
E3:1683102	C	T	MAD1L1	ENSFCAG00000005389	ENSFCAT00000005390	missense variant	MODERATE
E3:2251003	G	A	IQCE	ENSFCAG00000025676	ENSFCAT00000027142	missense variant	MODERATE
					ENSFCAT00000036337	missense variant	MODERATE
E3:2304718	C	T	BRAT1	ENSFCAG00000031391	ENSFCAT00000032068	missense variant	MODERATE
E3:2312083	G	A	BRAT1	ENSFCAG00000031391	ENSFCAT00000032068	missense variant	MODERATE
E3:2503530	G	A	CARD11	ENSFCAG00000008561	ENSFCAT00000008563	missense variant	MODERATE
					ENSFCAT00000036684	missense variant	MODERATE
					ENSFCAT00000061721	missense variant	MODERATE
E3:5354310	T	C	EIF2AK1	ENSFCAG00000008465	ENSFCAT00000024848	intron variant	MODIFIER
			ANKRD61	ENSFCAG00000027842	ENSFCAT00000033423	missense variant	MODERATE
E3:7130119	G	C	n/a	ENSFCAG00000023135	ENSFCAT00000046678	missense variant	MODERATE
E3:7878324	C	T	ZAN	ENSFCAG00000006851	ENSFCAT00000006853	missense variant	MODERATE
					ENSFCAT00000054763	missense variant	MODERATE
E3:7878456	A	G	ZAN	ENSFCAG00000006851	ENSFCAT00000006853	missense variant	MODERATE
					ENSFCAT00000054763	missense variant	MODERATE
E3:15165314	G	T	n/a	ENSFCAG00000008708	ENSFCAT00000008710	intron variant	MODIFIER
					ENSFCAT00000034285	intron variant	MODIFIER
					ENSFCAT00000053064	upstream gene variant	MODIFIER
					ENSFCAT00000063874	missense variant	MODERATE
E3:20493402	G	A	PRSS53	ENSFCAG00000002602	ENSFCAT00000002602	missense variant	MODERATE
			ZNF668	ENSFCAG00000008954	ENSFCAT00000008956	intron variant	MODIFIER
			ZNF646	ENSFCAG00000021918	ENSFCAT00000027839	downstream gene variant	MODIFIER
F1:17988451	T	C	CENPL	ENSFCAG00000013044	ENSFCAT00000013048	missense variant	MODERATE
F1:20764018	C	T	ASTN1	ENSFCAG00000006473	ENSFCAT00000006475	missense variant	MODERATE
					ENSFCAT00000039511	missense variant	MODERATE
					ENSFCAT00000053463	missense variant	MODERATE
F1:23027052	A	C	SOAT1	ENSFCAG00000007880	ENSFCAT00000007882	intron variant	MODIFIER
					ENSFCAT00000060726	intron variant	MODIFIER
			n/a	ENSFCAG00000036745	ENSFCAT00000047543	missense variant	MODERATE
F1:23028528	G	C	SOAT1	ENSFCAG00000007880	ENSFCAT00000007882	intron variant	MODIFIER
					ENSFCAT00000060726	intron variant	MODIFIER
			n/a	ENSFCAG00000036745	ENSFCAT00000047543	missense variant	MODERATE
F1:28499581	C	T	CACNA1E	ENSFCAG00000015444	ENSFCAT00000015448	missense variant	MODERATE
					ENSFCAT00000032756	missense variant	MODERATE
					ENSFCAT00000047607	missense variant	MODERATE
F1:30078275	C	T	CEP350	ENSFCAG00000014835	ENSFCAT00000014843	missense variant	MODERATE
F1:59343562	C	G	IARS2	ENSFCAG00000002597	ENSFCAT00000002597	missense variant	MODERATE
			BPNT1	ENSFCAG00000026600	ENSFCAT00000045775	upstream gene variant	MODIFIER
F1:60046003	C	G	HLX	ENSFCAG00000034331	ENSFCAT00000039455	missense variant	MODERATE
F1:61342644	C	T	HHIPL2	ENSFCAG00000002257	ENSFCAT00000002257	missense variant	MODERATE
F1:62240122	G	A	ALDH9A1	ENSFCAG00000004312	ENSFCAT00000004313	missense variant	MODERATE
F1:68196401	G	A	CD1B	ENSFCAG00000008648	ENSFCAT00000008650	missense variant	MODERATE
F1:69370149	G	A	BCAN	ENSFCAG00000006557	ENSFCAT00000006559	missense variant	MODERATE
					ENSFCAT00000058402	missense variant	MODERATE
F1:69629361	A	G	TMEM79	ENSFCAG00000011787	ENSFCAT00000011789	missense variant	MODERATE
			GLMP	ENSFCAG00000011788	ENSFCAT00000011790	downstream gene variant	MODIFIER
					ENSFCAT00000058515	downstream gene variant	MODIFIER
			SMG5	ENSFCAG00000011783	ENSFCAT00000050065	upstream gene variant	MODIFIER
F1:69787409	G	A	RAB25	ENSFCAG00000011775	ENSFCAT00000011777	missense variant	MODERATE
			LAMTOR2	ENSFCAG00000028596	ENSFCAT00000023659	downstream gene variant	MODIFIER
					ENSFCAT00000043261	downstream gene variant	MODIFIER
F1:70279561	G	T	RUSC1	ENSFCAG00000024900	ENSFCAT00000026005	missense variant	MODERATE
			RUSC1	ENSFCAG00000024900	ENSFCAT00000055419	upstream gene variant	MODIFIER
			FDPS	ENSFCAG00000039350	ENSFCAT00000034725	downstream gene variant	MODIFIER
F1:70502868	G	A	DCST1	ENSFCAG00000018847	ENSFCAT00000018172	missense variant	MODERATE
					ENSFCAT00000058210	missense variant	MODERATE
			DCST2	ENSFCAG00000040157	ENSFCAT00000053006	synonymous variant	LOW

F1:70503591	G	A	DCST1	ENSFCAG00000018847	ENSFCAT00000018172	upstream gene variant	MODIFIER
					ENSFCAT00000058210	upstream gene variant	MODIFIER
			DCST2	ENSFCAG00000040157	ENSFCAT00000053006	missense variant	MODERATE
F1:70932845	T	C	IL6R	ENSFCAG00000026747	ENSFCAT00000028335	missense variant	MODERATE
					ENSFCAT00000057276	missense variant	MODERATE
			RF00001	ENSFCAG00000024626	ENSFCAT00000031166	upstream gene variant	MODIFIER
F2:81054785	C	T	COL22A1	ENSFCAG00000040466	ENSFCAT00000056098	missense variant & splice region variant	MODERATE
F2:81202027	C	T	COL22A1	ENSFCAG00000040466	ENSFCAT00000056098	missense variant	MODERATE
F2:82973708	T	C	SLC45A4	ENSFCAG00000010846	ENSFCAT00000010851	missense variant	MODERATE
F2:83794075	C	T	TSNARE1	ENSFCAG00000010752	ENSFCAT00000010755	missense variant	MODERATE
					ENSFCAT00000043081	missense variant	MODERATE
X:10709501	C	T	OFD1	ENSFCAG00000014870	ENSFCAT00000014879	missense variant	MODERATE
					ENSFCAT00000042140	missense variant	MODERATE
					ENSFCAT00000062574	missense variant	MODERATE
X:10709512	G	T	OFD1	ENSFCAG00000014870	ENSFCAT00000014879	missense variant	MODERATE
					ENSFCAT00000042140	missense variant	MODERATE
					ENSFCAT00000062574	missense variant	MODERATE
X:26130933	A	G	NR0B1	ENSFCAG00000026851	ENSFCAT00000023728	missense variant	MODERATE
X:39321509	T	C	EFHC2	ENSFCAG00000007765	ENSFCAT00000007767	missense variant	MODERATE
X:104748999	G	C	TENM1	ENSFCAG00000008355	ENSFCAT00000008357	missense variant	MODERATE
					ENSFCAT00000046755	missense variant	MODERATE
					ENSFCAT00000066641	missense variant	MODERATE

Table 2. A list of variants unique to the proband that were called moderate impact by VEP and the effect in all associated transcripts.
